# The Bacillus of Plague

**Published:** 1897-05

**Authors:** 


### THE BACILLUS OF PLAGUE.

The report of Dr. H. T. Wilson, of the Hoagland Laboratory, who for some time past has been experimenting with plague microbes at the request of Commissioner Emery, the head of the Brooklyn Health Department, has just been made public. The bacilli came from Yepin, China, and were sent by Dr. W. F. Arnold, of the United States Navy, who is now attached to the Pacific Squadron. In order to test their activity he first inoculated several rabbits, all of whom rapidly succumbed to the disease originating from them. Test-tubes containing cultures of the bacilli in bouillon were then subjected to varying degrees of temperature, and it was found that up to 58° Centigrade the bacilli flourished and multiplied. At 59° their vigor became impaired, and at 60°, or about 120° Fahrenheit, their life became extinct. This relationship to heat reminds us that epidemics of the plague have not been observed south of 19° of latitude north. In the case of bits of paper, cloth and other material infected with the bacilli and then placed in a dry closet, the result was the same the germs not being able to survive a temperature of 60° C., or higher. As a result of his investigations Dr. Wilson concludes that the death-point is one or two degrees higher than that of the bacteria of this class of disease in general, and that unlike cholera germs, sunlight and dryness can not be relied upon to limit the life of this bacillus. He recommends that rags, nails, ballast and general merchandise coming from infected ports should be subjected, at either the port of departure or the port of entry, to a thorough system of disinfection. He has made experiments only with carbolic acid, and with this agent he has found that an exposure for two hours to a one per cent. solution suffices to destroy the life of the bacillus.—*Boston Medical Surgical Journal.*

